# Amino acids as modulators of the European seabass, *Dicentrarchus labrax*, innate immune response: an *in vitro* approach

**DOI:** 10.1038/s41598-017-18345-3

**Published:** 2017-12-21

**Authors:** Rita Azeredo, Cláudia R. Serra, Aires Oliva-Teles, Benjamín Costas

**Affiliations:** 1 Centro Interdisciplinar de Investigação Marinha e Ambiental (CIIMAR), Universidade do Porto, Terminal de Cruzeiros do Porto de Leixões, Avenida General Norton de Matos s/n, 4450–208 Matosinhos, Portugal; 2 Departamento de Biologia, Faculdade de Ciências da Universidade do Porto (FCUP), Rua do Campo Alegre s/n, Ed. FC4, 4169–007 Porto, Portugal; 3 Instituto de Ciências Biomédicas Abel Salazar, Universidade do Porto (ICBAS-UP), Rua de Jorge Viterbo Ferreira 228, 4050–313 Porto, Portugal

## Abstract

Teleost innate immune system is a most developed and powerful system in which fish highly rely throughout their lives. Conditions in aquaculture farms are particularly prone to disease, thus, health and welfare ensuring strategies are an urgent call to which nutrition is gradually becoming a most regarded achievement tool. This study intended to evaluate different amino acids’ effect on immune-related mechanisms as well as their potential as enhancers of European seabass, *Dicentrarchus labrax*, leucocyte functioning. To achieve these goals, primary cultures of head-kidney leucocytes were established and kept in amino acid (glutamine, arginine, tryptophan or methionine) supplemented culture media in two doses. The effects of amino acids treatments were then evaluated after stimulation with either *Vibrio anguillarum* or *Vibrio anguillarum* lipopolysaccharides by measuring nitric oxide production, extracellular respiratory burst, ATP and arginase activities, and expression of immune-related genes. Glutamine, arginine and tryptophan showed to be particularly relevant regarding cell energy dynamics; arginine and tryptophan supplementation also resulted in down-regulation of important immune-related genes. Immune responses in cells treated with methionine were generally enhanced but further studies, particularly those of enzymes activity, are essential to complement gene expression results and to better understand this nutrient’s immune role in fish.

## Introduction

In spite of being among the oldest vertebrates, teleosts are able to mount both innate and adaptive immune responses. While the latter are known to be less sophisticated than responses seen in higher vertebrates, the innate immune response is remarkably developed and powerful^1^, and fish highly rely on it^1,2^. Regardless of organism phylogeny, innate immune mechanisms are orchestrated by a great variety of cells and effector intermediates that are absent in specific immune responses^3^. Such feature grants the host with innate immune defences that, though unspecific, are able to react against a wide range of pathogens^4^. Although diseases are considered part of normal biological processes in fish, they may become a serious problem in aquaculture. Hence, as fish farming is a fast growing industry there is an urgent need to investigate and control fish diseases, as well as deeply understanding fish immunity.

It is now widely accepted that nutritional approaches are essential to alleviate diseases among farmed aquatic animals^5^. In particular, immune-nutritional strategies have been studied in order to highlight the importance of individual amino acids (AA) as nutraceutics for farmed fish. Glutamine, for instance, is a key source of energy for leucocytes^6^. During cell immune activation, glutamine is converted in alpha-ketoglutarate that fuels the Krebs cycle, providing additional energy to sustain immune function. *In vivo* and *in vitro* experiments with arginine demonstrated its involvement on immune regulation, cell proliferation and neuro-endocrine mechanisms, but its practical health implications in farmed fish are still controversial^7–10^. Arginine is precursor of nitric oxide, a potent bactericidal agent of the pro-inflammatory phase. It can also be converted in ornithine which, in turn, is the starting point for polyamine biosynthesis, essential players during cell proliferation. Tryptophan is involved in immune tolerance mechanisms mediated by its metabolites, following the enzymatic activity of indoleamine 2,3-dioxygenase in leucocytes^11,12^. The importance of tryptophan during dendritic cells activation has also been demonstrated^13^. Methionine supplementation of feeds affected fish immune status, as observed by increased leucocyte numbers^14^ and enhanced humoral immune response^14,15^. S-adenosylmethionine is an aminopropyl donor during polyamine turnover and a methyl donor that might change gene expression patterns^16^. Methionine might also indirectly affect the antioxidant capacity by providing cysteine to glutathione synthesis. Nonetheless, few studies were centred on the exact pathways through which AA mediate their immune effects.

One of the main players of innate response, particularly in inflammatory processes, is the phagocyte. Phagocytes are differentiated leucocytes (mostly neutrophils and macrophages) able to engulf and digest other cells (pathogens or self-apoptotic or necrotic cells). Phagocytes are directly involved in both induction and resolution of inflammation via paracrine and autocrine signalling^17^ by producing pro- and anti-inflammatory mediators. However, particularly in macrophages, such response only happens upon their activation/differentiation into mature cells that phenotypically present the required machinery. Based on stimuli that triggers different activation states, macrophages are classified in four groups (as recently reviewed by Forlenza and colleagues^18^): innate activated macrophages (triggered by microbial stimulus alone); classically activated macrophages (M1, resulting from synergistic effect of both microbial stimulus and IFNγ); alternatively activated macrophages (M2, induced by IL4 and IL13); and regulatory macrophages (activated by microbial stimulus, immune complexes, and IL10).

This study will focus only on innate activated macrophages, which are a valuable instrument in *in vitro* studies focused on the innate immune response^19–22^. Moreover, primary leucocyte cultures are useful tools in functional studies as they allow to evaluate responses under well controlled conditions and without the interference of external and unpredictable factors. In addition, it allows the evaluation of important cellular responses to bacteria or bacterial antigens and the assessment of signalling pathways eventually affected by treatments.

Antigens, such as lipopolysaccharides (LPS) from *E*. *coli*, are currently the most used immune response-triggering stimuli due to feasibility and commercial availability. However, though LPS easily elicits innate activation of mammal macrophages^23–25^ this is not the case in fish. Fish immune cells seem to be considerably less susceptible to LPS than mammals and much higher concentrations have to be administered (i.e. µg ml^−1^) for a response to be detected, apparently determined by different receptor-mediated recognition^26–28^. Presence of residual peptidoglycans or bacterial DNA in impure LPS preparations are believed to be the structures recognized by fish leucocytes, therefore responsible for inducing an immune response^29^.

We hereby present an *in vitro* study on the innate immune response of HKL against *Vibrio anguillarum* (*Vang*) or *Vibrio anguillarum* LPS (vaLPS), where we evaluated the modulatory effects of AA on immune-related signalling pathways. We also aimed to evaluate whether each AA surplus is able to improve the immune response in innate activated macrophages.

## Results

HKL were exposed to AA treatments by supplementing Leibovitz L-15 culture medium with each AA to the following final concentrations: _L_-glutamine (G1, 4.1 mM or G2, 5.1 mM), _L_-arginine (A1, 5.7 mM or A2, 7.2 mM), _L_-tryptophan (T1, 0.2 mM or T2, 0.25 mM) and _L_-methionine (M1, 1 mM or M2, 1.25 mM). A control group was included without addition of AA, henceforth referred as L-15 group.

### Nitric oxide

**Figure 1:**
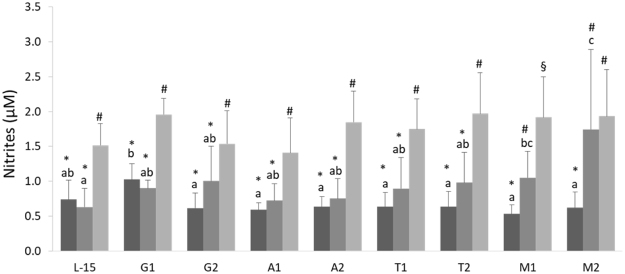
Nitric oxide content measured as total nitrites in the supernatant of HKL subjected to AA treatments under CTRL (), vaLPS () or *Vang* () stimulation conditions, regardless time of incubation. The AA treatments depicted in the X-axis are as follows: L-15, control; G1, 4.1 mM _L_-glutamine; G2, 5.1 mM _L_-glutamine; A1, 5.7 mM _L_-arginine; A2, 7.2 mM _L_-arginine; T1, 0.2 mM _L_-tryptophan; T2, 0.25 mM _L_-tryptophan; M1, 1 mM _L_-methionine; M2, 1.25 mM _L_-methionine. Values represent means ± SD (n = 6 biological replicates). Different symbols stand for statistically significant differences between stimuli within the same AA treatment, regardless time of incubation. Different letters denote statistically significant differences between AA treatments, within the same stimulus (Multifactorial ANOVA; Tukey post-hoc test; P ≤ 0.05).

A complete description of results can be found as Supplementary Table S1, and Fig. 1 provides a selected subset of most relevant results, regarding the effects of AA surplus on nitric oxide (NO) production. For clarity, results for each AA are described separately below.

#### Glutamine

At 72 h of incubation, G1-treated cells showed higher NO production than L-15-treated HKL (Supplementary Table S1C). A time-dependent effect was denoted in cells treated with G2, in which NO was higher at 96 h than at 72 h (Supplementary Table S1C). When incubated with *Vang*, G1- and G2-treated cells produced higher amounts of NO than their CTRL and vaLPS-incubated counterparts (Fig. 1). Moreover, NO levels in G1-treated cells were higher than in every other AA treatment when held under CTRL conditions (i.e. no stimuli) (Fig. 1).

#### Arginine

NO production increased with time only in A2-treated cells (Supplementary Table S1C), but no differences were observed in NO production of A1- and A2- treated cells relatively to L-15-treated cells (Supplementary Table S1B). Compared to CTRL or vaLPS-stimulated cells, incubation with *Vang* augmented NO production in both A1 and A2 groups (Fig. 1).

#### Tryptophan

NO production increased with time in both tryptophan supplementations (Supplementary Table S1C), but was not higher than in the L-15-treated cells (Supplementary Table S1B). Furthermore, T1- and T2- treated cells incubated with bacteria synthetized more NO than the respective CTRL and vaLPS groups (Fig. 1).

#### Methionine

The highest NO levels were measured in M2-treated cells, which were higher than in L-15, A1, A2 and T1 groups at 96 h of incubation (Supplementary Table S1C). NO levels increased in time in both methionine-supplemented groups (Supplementary Table S1C).

An interactive effect was observed between vaLPS and methionine, as vaLPS-stimulated M1 and M2 cells presented higher NO production than vaLPS-stimulated L-15 HKL, as well as their methionine CTRL counterparts, respectively (Fig. 1). Moreover, regarding M2-treated cells, these levels were also higher than those measured in every other AA treatments. Finally, both M1 and M2 *Vang*-stimulated cells presented higher NO production than their CTRL counterparts (Fig. 1).

### Respiratory Burst

A complete description of results on respiratory burst can be found as Supplementary Table S2.

**Figure 2:**
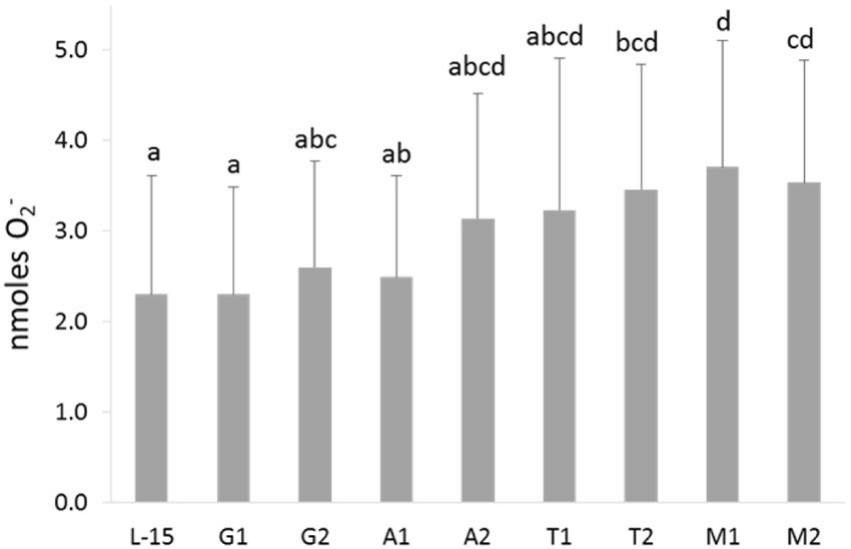
Extracellular superoxide anion (O_2_^−^) production in HKL subjected to the experimental treatments. The AA treatments depicted in the X-axis are as follows: L-15, control; G1, 4.1 mM _L_-glutamine; G2, 5.1 mM _L_-glutamine; A1, 5.7 mM _L_-arginine; A2, 7.2 mM _L_-arginine; T1, 0.2 mM _L_-tryptophan; T2, 0.25 mM _L_-tryptophan; M1, 1 mM _L_-methionine; M2, 1.25 mM _L_-methionine. Values represent means ± SD (n = 6 biological replicates). Different low case letters denote statistically significant differences between AA treatments, regardless stimulation or incubation time (Multifactorial ANOVA; Tukey post-hoc test; P ≤ 0.05).

Synthesis of O_2_^−^ presented the highest level at 4 h, regardless of stimuli or AA treatment (Supplementary Table S2B). Likewise, O_2_^−^ was higher in the CTRL group than in *Vang*-stimulated cells (Supplementary Table S2). Figure 2 provides a selected subset of the most relevant results, regarding the effect of AA treatments, regardless of stimulation or incubation time.

Cells treated with T2, M1 or M2 increased the production of O_2_^−^ compared to L-15 and G1 groups (Fig. 2). In M1-treated cells supernatant, O_2_^−^ levels were also higher than those observed in G2 and A1, while O_2_^−^ produced by M2-treated cells were increased compared to those of A1 group (Fig. 2).

### ATP

A complete description of results on extracellular ATP concentration in the supernatant of cells subjected to the different treatments can be found as Supplementary Table S3.

**Figure 3:**
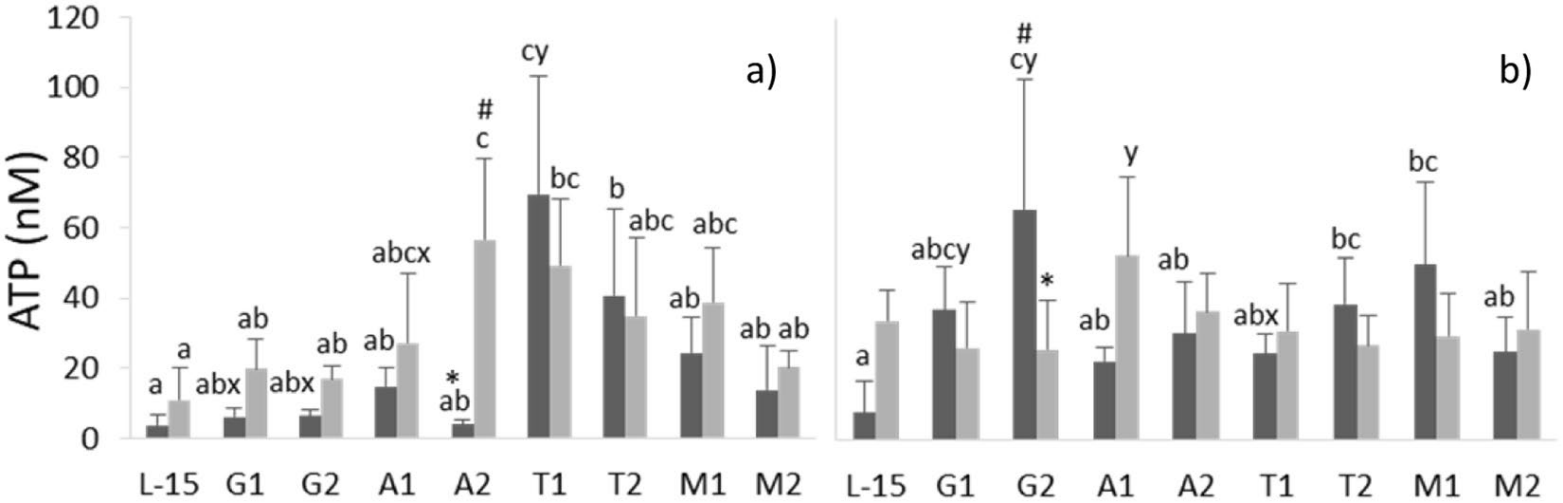
Extracellular ATP concentration in the supernatant of HKL subjected to the experimental treatments for 4 (**a**) or 24 h (**b**) under CTRL () or vaLPS () conditions. The AA treatments depicted in the X-axis are as follows: L-15, control; G1, 4.1 mM _L_-glutamine; G2, 5.1 mM _L_-glutamine; A1, 5.7 mM _L_-arginine; A2, 7.2 mM _L_-arginine; T1, 0.2 mM _L_-tryptophan; T2, 0.25 mM _L_-tryptophan; M1, 1 mM _L_-methionine; M2, 1.25 mM _L_-methionine. Values represent means ± SD (n = 6 biological replicates). * and ^#^ stand for statistically significant differences between stimuli within the same AA treatment and time of incubation. Different low case letters denote statistically significant differences between AA treatments within the same stimuli and incubation time. x and y stand for statistically significant differences between incubation time, within the same AA treatment and stimuli (Multifactorial ANOVA; Tukey post-hoc test; P ≤ 0.05).

Figure 3 provides a selected subset of the most relevant results, regarding extracellular ATP concentration in the supernatant of HKL incubated with AA treatments and stimulated with vaLPS for 4 or 24 h. For clarity, results for each AA are described separately below.

#### Glutamine

The highest ATP levels were produced by G2-treated cells incubated for 24 h in CTRL conditions which were higher than those of L-15, A1, A2, T1 and M2 groups, under the same conditions (Fig. 3b). An increase in ATP production with time was observed both in G1- and G2 groups under control conditions, but not in stimulated cells.

#### Arginine

ATP levels in stimulated A2-treated cells, but not in A1-treated cells, were higher than in their CTRL counterparts incubated for 4 h, as well as in stimulated L-15, G1, G2 and M2 cells in the same conditions (Fig. 3a). Moreover, ATP levels increased with time in the supernatant of stimulated A1-treated monolayers (Fig. 3).

#### Tryptophan

Both tryptophan supplementation levels improved ATP production particularly in CTRL HKL incubated for 4 h, compared to the L-15 group (Fig. 3a). At 4 h of incubation, ATP in non-stimulated T1-treated cells supernatant was also higher than all the other AA treatments under the same conditions (Fig. 3a). Moreover, T1 treatment led to augmented ATP production in cells stimulated with vaLPS for 4 h, compared to L-15-treated cells (Fig. 3a). In non-stimulated T2-treated cells, ATP production was higher than that produced by L-15 cells incubated for 24 h (Fig. 3b).

#### Methionine

ATP production in M1-treated cells was higher than in L-15 group when incubated in CTRL conditions for 24 h (Fig. 3b).

### Arginase Activity

The main purpose of measuring arginase activity was to understand the metabolic fate of arginine in innate-activated HKL, in parallel observation of NO synthesis. We also seek to find out whether it is dose-dependent. A complete description of results can be found as Supplementary Table S4.

Arginase activity was not affected by any arginine treatment, though a general increase with time was denoted (Supplementary Table S4B).

### Gene expression

A complete description of results on gene expression can be found as Supplementary Table S5.

Gene expression of *cox2* was down-regulated with time, regardless of cells being stimulated or not and independent of AA treatments, whereas it increased with the addition of vaLPS to cell medium (Supplementary Table S5B).

#### Glutamine

G2-treated cells led to up-regulation of *cox2* compared to L-15, A1, A2, T2 and M2 groups, regardless of time or stimulation, while in G1-treated cell it was only higher than A1, A2, T2 and M2 groups (Supplementary Table S5B). Though not statistically significant, *mif* mRNA levels were the highest in both glutamine treatments particularly when stimulated with vaLPS for 4 h. *tgfβ* was up-regulated in G1-treated cells compared to A1, A2, T2, M1 and M2 groups but only when incubated for 4 h (Supplementary Table S5B). On the other hand, comparatively to L-15-treated cells, *amd* and *odc* expression was inhibited in G1- and in G2-treated cells incubated for 24 h (Supplementary Table S5B). Also compared to L-15-treated cells, *odc* expression was inhibited in both glutamine supplementation levels both in CTRL and vaLPS-stimulated groups (Supplementary Table S5C). *sat* gene expression increased in time in both glutamine supplementations but no further effects were observed.

#### Arginine

Relatively to L-15-treated cells, expression of *il1β* was inhibited in unstimulated A2-treated HKL but was higher in both vaLPS-stimulated arginine supplementations than in their CTRL counterparts (Supplementary Table S5C). *cox2* gene expression was inhibited in A2-treated cells, regardless time of incubation or stimulation (Supplementary Table S5B). A decreasing trend was observed in both *mif* and *tgfβ* mRNA levels but it was devoid of statistical significance. *amd* and *odc* expression were down-regulated only at 24 h, in A1 treated-cells and in both A1- and A2-treated cells, respectively (Supplementary Table S5B). While *odc* was down-regulated in both unstimulated arginine supplementations, it was not significantly altered upon vaLPS stimulation (Supplementary Table S5C). In contrast, when incubated with vaLPS for 24 h, *sat* gene expression increased in A1- and A2- stimulated cells compared to their CTRL groups, an effect not observed in the other AA treatments or L-15-treated cells (Supplementary Table S5A).

#### Tryptophan

Lower expression of *odc* was observed at 24 h of incubation in T1-treated cells (Supplementary Table S5B), as well as in both tryptophan supplementations treatments in CTRL conditions (Supplementary Table S5C). However, in stimulated cells, *odc* down-regulation was only detected in T1-treated (Supplementary Table S5C). A non-significant decreasing trend was denoted in gene expression patterns of *il1β*, *cox2*, *tgfβ*, and *amd* in both tryptophan treatments (Supplementary Table S5B).

#### Methionine

Both *il1β* and *cox2* were down-regulated in M2-treated cells, though *il1β* inhibition occurred solely in CTRL conditions (Supplementary Table S5B and C, respectively). *mif* gene expression was up-regulated from 4 to 24 h in vaLPS-stimulated M2-treated cells (Supplementary Table S5A) whereas *odc* expression was down-regulated by both methionine treatments in cells incubated for 24 h (Supplementary Table S5B). Such inhibitory effect was observed in both CTRL and vaLPS-stimulated conditions. *amd* gene expression was also inhibited in M1-treated cells but only when incubated for 24 hours while M2-treated cells showed higher *amd* mRNA levels at 24 h than at 4 h of incubation (Supplementary Table S5B). *sat* was up-regulated at 4 h in vaLPS-stimulated M1-treated cells but only relatively to T2-treated HKL under the same conditions (Supplementary Table S5A).

## Discussion

An *in vitro* approach was carried out to evaluate possible effects of AA supplementation and the response of HKL upon immune stimulation over time. The expression of important immune-related genes was measured and indicators of cellular immune response were quantified.

The panel of genes selected to unveil possible AA modulatory effects was carefully assembled so that it would illustrate both innate immune mechanisms and polyamine biosynthesis mediated by innate activated macrophages. Unfortunately, variability of gene expression data was very high, not allowing to clearly unravel presumed effects, but just trends without statistical significance.

The present study showed that cell stimulation triggers the innate immune response of European seabass HKL, either cultured in L-15 alone or L-15 supplemented with individual AA. A general increase of NO parallel to upregulation of *cox2*, *il1β* and *tgfβ* genes point at modulatory effects that go beyond gene expression level. Immune cells activation is necessarily accompanied by increased NO production, as observed in the present study and several other studies in teleost^18,30,31^. Long incubation periods are necessary for the assessment of reactive nitrogen species as their concentrations are on the µM range. In this study, NO concentration was particularly low, but this was to be expected, as intensity of NO production seems to be species specific^19,32,33^. Moreover, each immune mechanism that is triggered implies an increased demand for energy to sustain the immune response. Extracellular ATP is believed to be a crucial mechanism of cell to cell communication during immune responses and extremely elevated concentrations (>100 µM) are indicative of cell death^34^. During inflammatory conditions, extracellular increasing levels of ATP are perceived by the cells through purinergic receptors promoting chemotaxis and interleukin 1 production in myeloid cells^35,36^. Accordingly, in the present study ATP production was observed to increase when cells were stimulated, though such increasing trend was more evident at 4 h of incubation. The observed decrease in superoxide anion production in stimulated cells compared to CTRL groups was probably related to exhaustion of respiratory burst, as already reported by Hardie *et al*.^37^.

Up-regulation of inflammatory genes is to be expected shortly after an immune response is triggered^7,38,39^. As mediator of prostaglandins biosynthesis, *cox2* is considered a key inflammatory signal^9,40^, together with other pro-inflammatory mediators such as *il1β* and tumour necrosis factor alpha (TNFα), and their expression generally increase upon immune stimulation^28,41^. Accordingly, both *il1β* and *cox2* were modulated in this study, their gene expression increasing in vaLPS-stimulated cells and decreasing over time irrespective of AA treatments. In contrast, while similarly higher in vaLPS-stimulated cells, *tgfβ* mRNA expression increased in time. *tgfβ* is a potent negative regulator of haematopoiesis, modulating proliferation, differentiation, and function of several cell types^42^. Hence, it can be considered an anti-inflammatory intermediate that counteracts other earlier cytokines actions as the immune response develops, thus controlling the inflammatory process in a later stage. *mif* has been previously characterized as a constitutively expressed gene in immune cells, being stored as preformed protein and promptly released upon immune stimulation with LPS^43^. This fact might help explain the absence of a significant up-regulation in stimulated cells. Still, recent findings demonstrated the responsiveness of this gene transcription to microbial products, thereby promoting inflammatory responses^44^. Differently from pro-inflammatory cytokines, highest *mif* expression levels were not observed at 4 h of vaLPS incubation, but this may be explained by the immediate translated protein availability.

HKL incubated in glutamine-supplemented media (i.e. G1 and G2) enhanced NO and energy production. NO is exclusively synthetized from arginine, but glutamine can be recycled to arginine via citrulline during the urea cycle in mammals^45^. Hence, besides being a by-product of arginine upon NO synthesis, citrulline is also the primary endogenous source of arginine and evidences have showed the same to occur in lower vertebrates such as teleost fish, though at much lower rates^46,47^. In channel catfish, *Ictalurus punctatus*, activated macrophages, medium citrulline concentration decreased below detection levels while NO production was improved when arginine and glutamine were simultaneously added^48^. Hence, one could attribute an increased NO production to glutamine-associated nitrogen pool as it has been done in mammals^49^.

In immune cells such as lymphocytes or macrophages, glutamine utilization rates are even higher than those of glucose^50^. Indeed, these cells take advantage of glutamine availability when energy demands increase due to immune stimulation^49,51^. Being so, glutamine studies have been focused on its role as energy source^52,53^. Glutamine addition to HKL monolayers might have benefited these cells in terms of energy balance, as supported by the enhanced ATP production, promoting the synthesis of proinflammatory signals (*cox2*) and increasing the efficiency of immune mechanisms such as NO production. It would also be expected that glutamine treatment resulted in increased respiratory burst, as previously denoted by Cheng and co-workers^54,55^. Differently, superoxide anion production was not affected by glutamine supplementation to the media. A possible explanation would be that glutamine, being a glutathione precursor, could have increased glutathione pool^56–58^, which in turn would contribute to reduce levels of free radicals such as superoxide anion. Nonetheless, this hypothesis would need to be confirmed by glutathione quantification analysis, and this should be taken into account in future studies concerning glutamine and the immune response.

Arginine, besides its obvious and direct role in innate immune response as NO sole precursor, is also determinant in designating T cell function in differentiated myeloid cells^59,60^. In this work, however, arginine supplementation to HKL monolayers did not affect NO synthesis nor arginase activity. Both inducible nitric oxide synthase (iNOS) and arginase activities are cytokine-induced but their expression occurs in differently activated myeloid cells^18^. While iNOS is typically expressed by innate- or classically- activated macrophages, arginase activity is a trait of alternatively-activated macrophages^61^. Because these two macrophage pathways have opposite and competing actions, i.e. iNOS is pro-inflammatory and arginase down-regulates inflammation, expression patterns should be opposite and consequently so should NO production and arginase activity. In accordance, and in spite of absence of an AA effect, arginase activity was higher at 24 h compared to levels at 4 h which, supported by *tgfβ* transcript data, seems to indicate that anti-inflammatory processes start to develop later on in the immune response.

The absence of significant effects on NO production could be related to arginine supplemented dose and incubation time, since there is a clear time effect. In channel catfish, peritoneal macrophages incubated in an arginine-supplemented medium produced much higher NO amounts than control cells in response to 96 h of LPS incubation^48^. In the present experiment, arginine concentration was much higher than that used in the aforementioned study. Being kept in such conditions for 120 h, arginine-treated macrophages might have suffered from earlier toxic effects of NO that impaired cell function, as suggested by Mills^62^, hence no further increase was observed at the end of LPS incubation period. Still, additional and earlier measuring points are required to confirm this hypothesis.

In spite of *amd* expression patterns were lower in A1-treated HKL (an observation transversal to most AA treatments), such inhibition was not present in cells incubated with A2. Moreover, differently from other groups, arginine supplementation did not inhibited *odc* in stimulated cells. This, together with levels of *sat* mRNA in 24 h-vaLPS-stimulated cells being highest upon arginine surplus, may suggest that activation of these cells might have promoted polyamine turnover in a dose- and time-dependent manner. Andersen *et al*.^63^ tested the effect of doubling arginine concentration on Atlantic salmon, *Salmo salar*, hepatocytes primary cell cultures and observed no alteration on *sat* mRNA levels. However, the abundance of the translated enzyme was highest in cells of individuals fed arginine-supplemented diets. Our results under CTRL conditions are in accordance with those of Andersen and colleagues, but our data on the presence of an immune stimulus evidences the potential of using arginine to boost polyamine turnover. Naturally, gene expression analysis per se is not determinant for the evaluation of final protein abundance, but differences observed between arginine group and L-15 or other AA treatments, at least points out the most likely pathway through which these macrophages are metabolizing arginine. The almost unaltered NO production and respiratory burst response, which were the lowest responses among all AA treatments, might further support that polyamine biosynthesis is the preferred metabolic pathway of macrophages treated with arginine. Accordingly, both pro-inflammatory genes *il1β* and *cox2* were down-regulated in cells incubated with the highest arginine surplus. Despite of conflicting information, previous *in vivo* and *in vitro* studies on arginine immune modulation describe similar inhibitory effects that generally impair several immune mechanisms^7,8,64^.

In the present study, HKL stimulated with vaLPS in combination with arginine treatment raised ATP synthesis, in contrast to any other treatment which reduced ATP production upon immune stimulation. This suggests that arginine could also have been used for energy production besides NO production. Cheng and co-workers^65^ recently reviewed the importance of the cross-talk between immune response and metabolism, not only at the organism level, but also at individual cells level. In fish, high levels of ATP can be generated during partial AA catabolism and, particularly, high ATP yields in the conversion of arginine to alanine have been observed^66^. Alanine would further be advantageous since it forms a useful carrier of AA carbon to further metabolism elsewhere^67^. It is thus tempting to speculate that arginine catabolism was used to support an increased energy demand upon immune stimulation, supported by low superoxide anion and NO production in combination with poor arginase activation.

Indeed, energy demand must be compensated by intense ATP production, which in turn rely on adequate NAD+ supply. In the present study, at early phase of incubation and regardless of the presence of a stimulus, the highest ATP levels were observed in T1- and T2-treated HKL. In mammals, in the absence of niacin, tryptophan is the sole source of substrate for NAD+ production^12^, via the kynurenine pathway. Although kynurenine pathway is far from being completely established if fish, evidences of presence of key enzymes have been reported in fish head-kidney cells^68^. In our *in vitro* model, cells were incubated with Leibovitz’s L-15 medium, which already contains niacin, so the base level of this compound was the same for all AA treatments. So, comparatively to other AA treatments, the two tryptophan groups are expected to be benefited.

The focus of tryptophan involvement on the immune response has mostly been on the enzyme indoleamine 2,3-dioxygenase (IDO) which seems to be associated to regulation of T-cell function and thereby to immunosuppressive effects^12,69,70^. In accordance, inflammatory mediators mRNA levels were repeatedly (though not significantly) lower in T2-treated cells, suggesting that an inflammatory signal might have been impaired. However, while tryptophan failed to enhance NO production, extracellular superoxide anion levels were enhanced, which could be linked to improved cellular performance ensured by higher ATP production. Immunosuppression is mediated by 3-hydroxykynurenine, 3-hydroxyanthranilic acid and quinolinic acid, which are major downstream breakdown products of tryptophan catabolism known to inhibit immune cells proliferation and function^12^. *amd* is indirectly involved in polyamine biosynthesis, hence in cell proliferation, and was lower in both tryptophan supplementation levels. Altogether, observations seem to point out an attenuated inflammatory response caused by higher tryptophan availability. Future work on tryptophan and fish immune response should include the evaluation of IDO activity coupled with gene expression of dendritic and T-cell markers.

In this study, the most expressive modulatory effects were those induced by methionine supplementation, that boosted innate immune defences such as NO and superoxide anion production and enhanced ATP yields. One of the most remarkable effects of methionine immune modulation was the increase HKL reactivity to vaLPS. While M1-treated cells already showed higher vaLPS-induced NO response than that of L-15-treated cells, the highest methionine supplementation elevated LPS-induced NO to concentrations as high as those seen against *Vang* itself. The low sensitivity of fish to LPS is associated to the pathogen recognition receptor TLR4, which is known to be activated by LPS in mammals but, when present, is believed to have different functions in teleosts^26,71^. Although methionine improves HKL immune response to vaLPS it does not necessarily mean that it modulates immune cells activation pathways, but it highlights the potential of methionine to strengthen cells immune response. This hypothesis is also supported by the improved ATP production and respiratory burst in these cells. Methionine was the most powerful AA at improving leucocytes oxygen radicals (both NO and superoxide anion production). As precursor of cysteine, a constituent of glutathione, methionine is expected to regulate cellular redox potential and, ultimately, the amount of free radicals such as superoxide anion. However, the ability of this AA to increase other non-directly related mechanisms (NO, ATP, gene expression) suggests that methionine might also indirectly modulate immune defences such as the respiratory burst, via methylation or polyamines biosynthesis pathway. As observed by Kuang *et al*.^15^, the activity of superoxide dismutase and other antioxidant enzymes in the head-kidney of juvenile Jian carp, *Cyprinus carpio var*. *Jian*, were reduced upon addition of graded levels of dietary methionine hydroxyl-analogue.

Unexpectedly, methionine down-regulated the expression of two important inflammatory genes, *il1β* and *cox2*. Such impairment was stronger in unstimulated HKL treated with the higher methionine level. *cox2* mRNA levels seemed to respond to methionine on a dose-response basis, as values were higher in M1-treated cells compared to L-15 cells but lower in M2-treated cells, especially upon vaLPS stimulation, although the difference was not statistically significant. Though much higher than non-treated cells, a similar behaviour was observed with superoxide and ATP production. Such dose-effect suggests that methionine might be beneficial within a certain range of concentrations, an issue in need of further research.

Polyamine turnover is fuelled by the transferring of aminopropyl groups, which enables polyamines interconversion^72^. Decarboxylated s-adenosylmethionine is the donor molecule for this interconversion and this is its sole role, with *amd* being enzyme mediating the decarboxylation. Previous data on methionine dietary supplementation clearly demonstrated the stimulatory effect of this AA on peripheral leucocyte proliferation and, consequently, on the improvement of innate humoral response^14^. In this study, methionine supplementation increased *amd* gene expression from 4 to 24 h of incubation, which was not observed in any other treatment. These results suggest that methionine immune modulation might be mediated by an improvement of polyamine biosynthesis, which in turn increases immune cell proliferation. However, as *odc* was conversely inhibited in both methionine-treated groups, this hypothesis requires additional research.

In summary, the present *in vitro* study highlighted relevant immunomodulatory effects of different AA on seabass HKL. HKL took advantage of glutamine, arginine, and tryptophan as energy sources that, yielding higher ATP amounts, seem to enhance these cells immune performance. Arginine seemed to potentiate macrophages acquisition of an immune suppressive phenotype, as supported by arginase activity increase over time and enhanced *sat* gene expression, which might indicate improved polyamine turnover. Tryptophan enhanced respiratory burst, but it also down-regulated inflammatory-related and polyamine-related gene expression which seems to point at the presence of immune-suppressive (IDO-mediated) mechanisms. Differently, methionine treatment seemed to improve cellular innate immune defences and it may also play a role on cell proliferation. Altogether, these results signal clear immunomodulatory effects of AA, which highlights the potential for the establishment of immune-nutritional strategies in aquaculture that need to be further investigated.

## Methods

### *Vibrio anguillarum* cell inactivation

The addition of viable bacteria to eukaryotic cell cultures necessarily involves nutrients consumption. Consequently, not only nutrients availability is compromised and AA treatments jeopardized, but cell medium pH is altered. Therefore, stimulating HKL with live bacteria would probably lead to a noxious *in vitro* environment that do not serve present study main goals. Hence, bacteria cultures were inactivated by UV-exposure. Since UV exposure inactivates bacteria by disrupting nucleic acids, integrity of the main structures is assured thus allowing normal antigen recognition by eukaryotic cells. *Vang* was kindly provided by Professor Alicia E. Toranzo (Departamento de Microbiología y Parasitología, Facultad de Biología, University of Santiago de Compostela, Spain) and previously isolated from gilthead seabream (*Sparus aurata*). Bacteria were first cultured for 24 h at 22 °C in selective medium thiosulfate citrate bile salts sucrose (TCBS) agar (VWR, Prolabo). Colonies were then inoculated into tryptic soy broth (TSB) supplemented with NaCl to a final concentration of 1% (w/v) and incubated overnight at 22 °C. Bacterial solution was inactivated by exposure to UV-light for 2 h preceded by alternating UV-light exposure for 10 min (2 min UV-light with 1 min interval), which proved to increase the efficiency of bacterial inactivation. Bacterial growth was not observed when UV-killed bacteria were plated in tryptic soy agar. Bacteria were then recovered by centrifugation at 3,500 rpm for 30 min and the pellet was then re-suspended in sterile phenol-red free Hank’s Balanced Salt Solution (HBSS) at final concentration of 1 × 10^7^ colony forming units (CFU) ml^−1^.

### *Vibrio anguillarum* lipopolysaccharides extraction and purification

*V*. *anguillarum* LPS was extracted by hot phenol-water according to the method described by Rezania *et al*.^73^ with slight modifications. One hundred ml of bacterial suspension (9 × 10^8^ CFU ml^−1^) was centrifuged at 10,000 × *g* for 5 min and washed twice with 0.15 M phosphate buffer saline (PBS) (pH = 7.2) containing 0.15 mM CaCl_2_ and 0.5 mM MgCl_2_. Pellets were re-suspended in 10 ml PBS and sonicated on ice for 10 min. To eliminate protein and nucleic acid contaminants, the samples were treated with proteinase K (100 μg ml^−1^; Roche, Mannheim, Germany) for one hour at 65 °C, followed by overnight incubation at 37 °C with RNase (40 μg ml^−1^; Roche) and DNase (20 μg ml^−1^; Roche) in presence of 1 μl ml^−1^ 20% MgSO_4_ and 4 μl ml^−1^ chloroform. Next, an equal volume of hot (65–70 °C) 90% phenol was added to the mixture followed by vigorous shaking at 65–70 °C for 30 min. After being cooled on ice, the mixture was transferred to 50 ml polypropylene tubes and centrifuged at 3,500 × *g* for 30 min. The supernatants were recovered and extra phenol phases were removed using 20 ml hot (65–70 °C) distilled water. Sodium acetate at 0.5 M final concentration and 10 volumes of 95% ethanol were added to the extracts to precipitate vaLPS by incubating overnight at −20 °C. Tubes were then centrifuged at 3,500 × *g*, 4 °C for 30 min and pellets were re-suspended in 1 ml distilled water, followed by extensive dialysis (SnakeSkin dialysis tubing of 10 K MWCO, Thermo Fischer Scientific) against distilled water at 4 °C. Purified vaLPS, without any residual phenol was lyophilized, re-suspended in PBS to a final concentration of 2 mg ml^−1^, and kept at −20 °C until use. Visualization was achieved by SDS-PAGE (12%) electrophoretic resolution of 20 µg purified vaLPS and consequent staining following the improved silver stain protocol described by Zhu *et al*.^74^.

### Fish and establishment of HKL primary cell cultures

European seabass (*Dicentrarchus labrax*) of 200 ± 50 g were obtained from a commercial fish farm. The animals were acclimatized to laboratory conditions for 3 weeks in a recirculation water system maintained at 18 °C and daily fed a commercial diet (Skretting, Spain). No clinical signs of disease or illness were observed in the animals.

HKL were isolated and maintained following Secombes^75^ methodology with some modifications. Briefly, European seabass head-kidney was aseptically removed and pushed through a 100 µm nylon mesh in Leibovitz L-15 medium (Gibco, Scotland, UK) supplemented with 2% foetal calf serum (FCS, Gibco), penicillin (Gibco; 100 IU ml^−1^), streptomycin (Gibco; 100 µg ml^−1^), gentamicin (200 µg ml^−1^) and heparin (Braun, 30 U ml^−1^). The cell suspension was then layered over a 31:45% Percoll column (Sigma) and centrifuged at 400 × *g* and 4 °C for 40 minutes. Cells were recovered from the Percoll gradient interface, loaded onto a new Percoll gradient and centrifuged again in the same conditions. Then, the cell band was washed three times in L-15 medium 2% FCS, heparin and antibiotics at 600 × *g* and 4 °C for 10 minutes. Cells were re-suspended in L-15 medium, 0.1% FCS and antibiotics, and both leucocyte viability and concentration were determined by the trypan blue exclusion method, using a Neubauer chamber. Cell suspension was then adjusted to 1 × 10 ^7^ cells ml^−1^ and plated in 96-well plates at 100 µl per well (for respiratory burst, NO, ATP assay and arginase activity measurements) or 24-well plates at 500 µl per well (for gene expression). Plates were incubated for 2 hours at 18 °C and non-adherent cells were then washed off with HBSS.

The experiments were approved by the Animal Welfare Committee of the Interdisciplinary Centre of Marine and Environmental Research and carried out in a registered installation (N16091.UDER) and were performed by trained scientists in full compliance with national rules and following the European Directive 2010/63/EU of the European Parliament and the European Union Council on the protection of animals used for scientific purposes.

### Experimental conditions

L-15 is a standard eukaryotic cell culture media which composition contains the amino acids considered in this study. Monolayers were kept for 24 h at 18 °C in L-15 10% FCS supplemented with each AA. AA concentrations were selected based on preliminary studies in which low supplementation doses (0.5 mM and 1 mM) did not significantly affect NO levels or respiratory burst. Hence, all AA were tested at 1 × or 1.5 × basal concentration to the following final concentrations: _L_-glutamine (G1, 4.1 mM or G2, 5.1 mM), _L_-arginine (A1, 5.7 mM or A2, 7.2 mM), _L_-tryptophan (T1, 0.2 mM or T2, 0.25 mM) and _L_-methionine (M1, 1 mM or M2, 1.25 mM). A control group without addition of AA, henceforth referred as L-15 group, was also included. HKL cultures were visually inspected and photographed (Olympus IX71).

Whenever possible, functional immune responses were evaluated following stimulation with *Vang* (added at a final concentration of 1 × 10^6^ CFU ml^−1^) as teleost leucocytes are more reactive to bacteria than to LPS alone^27,29^. However, for gene expression and ATP studies cell monolayers were only stimulated with vaLPS (10 µg ml^−1^) to avoid bacterial RNA and ATP contamination. NO production was tested using both stimuli, as preliminary tests in this parameter showed relevant results regarding AA and stimuli interactive effects. A control group with no stimulus (CTRL) was always included.

Incubation periods differed between analyses, as different responses have different timings. All parameters were measured at the end of 4 or 24 h of stimulation since most innate immune mechanisms are triggered upon stimulation and measurable soon afterwards. NO production was tested at the end of 72 h and 96 h, since it is not as yet measurable by this protocol at earlier time points, as shown in turbot, *Scophthalmus maximus*, macrophages after LPS exposition^32^.

Every treatment analyses were carried out in triplicate wells, and a total of six biological replicates were used for each analysed parameter.

### Nitric oxide

NO production was measured indirectly based on the Griess reaction previously described by^31^ and modified according to^32^. As NO is a very unstable molecule and is promptly converted into nitrite and further into nitrate, this method quantifies nitrites content in the supernatant of macrophage primary cell cultures. Following 24 h incubation with each AA treatment or L-15 alone, cell culture media was replaced by fresh solution containing either *Vang* or vaLPS. Then, plates were incubated at 18 °C for 72 or 96 h. Nitric oxide At the end of each incubation time, 50 µl of leucocytes supernatant were transferred into a new 96-well plate to which 100 µl of sulfanilamide (Sigma, 1% in phosphoric acid at 2.5%) and 100 µl N-naphthylethylene-diamine (Sigma, 0.1% in phosphoric acid at 2.5%) were added. Absorbance was read at 540 nm (Synergy HT, Biotek) and nitrites molar concentration was obtained against a sodium nitrite standard curve.

### Respiratory Burst

Prior to respiratory burst assessment, primary cell cultures were stimulated for 4 or 24 hours at 18 °C with *Vang* solution containing each AA treatment or L-15 alone. A control group with no stimulus was also included. Respiratory burst activity of HKL was measured based on the reduction of ferricytochrome C method for detection of extracellular O_2_^−^^75^. After each incubation time, leucocyte monolayers were firstly washed twice with HBSS. Then, 100 µl of ferricytochrome C solution (Sigma, 2 mg ml^−1^ in HBSS) with phorbol myristate acetate (PMA, Sigma, 10 µg ml^−1^) was added to the wells. To confirm the reaction specificity, wells containing ferricytochrome C, PMA and superoxide dismutase (SOD, Sigma, 300 uni ml^−1^) were included for each assay. Plates were incubated for 30 min at 18 °C, and the absorbance was measured at 550 nm (Synergy HT, Biotek). Optic density (OD) units were transformed into nmols of O_2_^−^ by multiplying by the conversion factor 15.87, as described by^76^.

### Arginase activity

Arginase activity was only measured in the lysate of HKL cultures supplemented with A1, A2 or in control conditions (L-15). Cells were incubated for 4 or 24 h at 18 °C with *Vang*. Then, cell monolayers were removed from each well (≈10^6^ cells ml^−1^) with cold HBSS without Ca^2+^ or Mg^2+^, and the cell suspension was centrifuged for 10 min at 1000 × *g* and 4 °C. HKL were then lysed for 10 minutes with 100 µl Tris-HCl solution (pH 7.4, 1 mM pepstatin A, 1 mM leupeptin, and 0.4% (w/v) Triton X-100). Afterwards, cell lysates were centrifuged at 13,000 × *g* and 4 °C, and the supernatant recovered. Arginase activity was then performed with the Arginase Activity Assay Kit (Sigma) according to manufacturer’s instructions. Briefly, 40 µl of supernatant was added to duplicate wells. Fifty µl of 1 mM urea standard solution and 50 µl water were also added to separate wells. Then, 10 µl of arginine substrate buffer was added to one of the sample wells and plates were incubated for 2 h at 37 °C. Afterwards, 200 µl of urea reagent was added to all wells to stop the reaction followed by the addition of 10 µl of arginine substrate buffer to the other sample well. Plates were incubated again for 60 min at room temperature and absorbance was measured at 430 nm (Synergy HT, Biotek). OD units were converted to units ml^−1^ according to manufacturer’s instructions.

### ATP assay

Stimulation of cells for the ATP production analysis was achieved by incubating cell monolayers with vaLPS for 4 or 24 h at 18 °C, with prior 24 h incubation with each AA treatment or L-15 alone. The ATP concentration in HKL cultures was measured with the ATP Determination Kit (Molecular Probes) following manufacturer’s indications. In summary, 10 µl of each well supernatant was transferred to a new plate containing 90 µl of standard reaction solution comprising dithiothreitol, D-luciferin and luciferase, and luminescence was read (Synergy HT, Biotek). Background luminescence values were subtracted and ATP concentration was calculated from a previously prepared ATP standard curve.

### Gene expression

Total RNA was extracted with NZYol reagent (NZYTech, Portugal) following manufacturer’s instructions, and resuspended in free nuclease water (NZYTech). RNA was quantified using Take 3 Microvolume Plate (Biotek) and samples were then treated with DNase using RQ1 RNase-free DNase kit (Promega) following manufacturer’s indications. The integrity of total RNA was assessed on denaturing agarose gels. Total RNA (600 ng) per sample was used for cDNA synthesis, which was performed using NZY First-Strand cDNA Synthesis Kit (NZYTech) according to manufacturer’s instructions.

A set of primers was chosen to evaluate immune-relevant gene expression profile. The chosen genes were: interleukin-1β (*il1β*), cyclooxygenase 2 (*cox2*), macrophage migration inhibitory factor (*mif*), and transforming growth factor β (*tgfβ*). To estimate polyamine synthesis modulation, three genes involved in this pathway as well as in AA metabolism were selected: s-adenosylmethionine decarboxylase 1 (*amd*), ornithine decarboxylase (*odc*) and diamine acetyltransferase 1 (*sat*). Primer sequences are listed in Table 1. Efficiency of each primer pair was determined by real-time PCR according to Pfaffl^77^. Quantitative PCR reactions were carried out in an Eppendorf Mastercycle ep realplex. Each reaction contained 1 µl of diluted cDNA (1:5 dilution) mixed with 10 µl of NZYSpeedy qPCR Master Mix and 0.4 µl (10 mM) of each specific primer, in a final volume of 20 µl. The thermal conditions used were 10 min at 95 °C of pre-incubation, followed by 40 cycles at 95 °C for 15 s and annealing temperature for 1 min. Melting curve analysis was performed to verify that no primer dimers were amplified. The expression of target genes was normalized using European seabass ubiquitin (*ubqt*) gene as housekeeping gene, since it was constitutively expressed independently of treatments. Fold change units were calculated by dividing the normalized expression values from different treatments by the normalized expression values of the respective controls.

**Table 1:** Forward and reverse primers for real-time PCR.

Gene name	Symbol	GenBank	*Eff* ^1^	AT^2^	Product length^3^	Primer sequence (5′-3′)
Interleukin 1 β	*il1β*	AJ269472.1	97.7	57	105	F AGCGACATGGTGCGATTTCT R CTCCTCTGCTGTGCTGATGT
Cyclooxygenase 2	*cox2*	AJ630649.1	81.3	60	160	F CATTCTTTGCCCAGCACTTCACC R AGCTTGCCATCCTTGAAGAGTC
Macrophage migration inhibitory factor	*mif*	FN582353	89.3	60	76	F GCTCCCTCCACAGTATTGGCAAGAT R TTGAGCAGTCCACACAGGAGTTTAGAGT
Transforming growth factor β	*tgfβ*	AM421619.1	105.4	55	143	F ACCTACATCTGGAACGCTGA R TGTTGCCTGCCCACATAGTAG
S-adenosylmethionine decarboxylase	*amd*	KM225770	99.3	57	63	F CTGACGGAACTTACTGGACCATC R CGAAGCTGACGTAGGAGAACT C
Ornithine decarboxylase	*odc*	KM225771	99.9	60	69	F GGGCTGTAGTTATGACACTGGCATCC R GCTGAATCTCCATCTTGCTTGCACAGT
Diamine acetyltransferase 1	*sat*	KM225772	91.6	63	55	F GCATCATCGCTGAAATCCAAGGAGAGAACA R CCAACCACCTTCAGGCCGTCACT
Ubiquitin	*ubqt*	FN565665	92.7	55	79	F TGCTCCCAATCCAGATGATCC R TGTCTCGATGGCGTTGCTT

^1^Efficiency of PCR reactions were calculated from serial dilutions of tissue RT reactions in the validation procedure.

^2^Annealing temperature (°C).

^3^Amplicon (nt).

### Statistical analysis

Statistical analyses were performed with STATISTICA (StatSoft, Inc. 2013, version 12) for WINDOWS. Results are expressed as means ± standard deviation. Homogeneity (Levene’s test) and normality were checked and, when necessary, outliers were removed and data were log-transformed before analysis. Data were analysed by multifactorial analysis of variance (ANOVA) with time, AA and stimulus as factors, and with p ≤ 0.05 as significance level chosen for rejection of the null hypothesis. A multiple-comparison Tukey HSD test was performed to identify differences between groups.

### Data availability

The datasets generated and analysed during the current study are available from the corresponding author on reasonable request.

## Electronic supplementary material

Supplementary File
