# Supplementary material for: Amino acids as modulators of the European seabass, Dicentrarchus labrax, innate immune response: an in vitro approach
Source: Sci Rep. 2017 Dec 21;7:18009. doi: 10.1038/s41598-017-18345-3 (PMC5740149; doi:10.1038/s41598-017-18345-3)
Supplement: Supplementary file 1 — Supplementary File [file 41598_2017_18345_MOESM1_ESM.doc]

**Amino acids as modulators of the European seabass, *Dicentrarchus labrax*, innate immune response: an *in vitro* approach**

Rita Azeredo^1, 2*^, Cláudia R. Serra^1^, Aires Oliva-Teles^1, 2^, Benjamín Costas^1,3*^

^1^Centro Interdisciplinar de Investigação Marinha e Ambiental (CIIMAR), Universidade do Porto,

Terminal de Cruzeiros do Porto de Leixões, Avenida General Norton de Matos s/n, 4450-208 Matosinhos, Portugal.

^2^Departamento de Biologia, Faculdade de Ciências da Universidade do Porto (FCUP), Rua do Campo Alegre s/n, Ed. FC4, 4169-007 Porto, Portugal.

^3^Instituto de Ciências Biomédicas Abel Salazar, Universidade do Porto (ICBAS-UP), Rua de Jorge Viterbo Ferreira 228, 4050-313 Porto, Portugal

***Corresponding authors:**

Rita Azeredo

Benjamín Costas

Email addresses: mleme@ciimar.up.pt; bcostas@ciimar.up.pt

Table S1 A. Nitric oxide content measured as total nitrites in the supernatant of European seabass HKL subjected to experimental conditions for 72 h or 96 h.

| AA | stimulus | time | NO *(*µM) |
| --- | --- | --- | --- |
| L-15 | CTRL | 72 | 0.54 ± 0.19 |
|  |  | 96 | 0.99 ± 0.05 |
|  | vaLPS | 72 | 0.49 ± 0.14 |
|  |  | 96 | 0.77 ± 0.30 |
|  | *Vang* | 72 | 1.33 ± 0.22 |
|  |  | 96 | 1.75 ± 0.27 |
|  |  |  |  |
| G1 | CTRL | 72 | 0.91 ± 0.23 |
|  |  | 96 | 1.17 ± 0.12 |
|  | vaLPS | 72 | 0.90 ± 0.12 |
|  |  | 96 | 0.90 ± 0.12 |
|  | *Vang* | 72 | 1.80 ± 0.14 |
|  |  | 96 | 2.12 ± 0.19 |
|  |  |  |  |
| G2 | CTRL | 72 | 0.43 ± 0.13 |
|  |  | 96 | 0.80 ± 0.06 |
|  | vaLPS | 72 | 0.67 ± 0.31 |
|  |  | 96 | 1.33 ± 0.44 |
|  | *Vang* | 72 | 1.21 ± 0.28 |
|  |  | 96 | 2.01 ± 0.17 |
|  |  |  |  |
| A1 | CTRL | 72 | 0.54 ± 0.06 |
|  |  | 96 | 0.63 ± 0.12 |
|  | vaLPS | 72 | 0.60 ± 0.06 |
|  |  | 96 | 0.81 ± 0.29 |
|  | *Vang* | 72 | 1.10 ± 0.32 |
|  |  | 96 | 1.94 ± 0.19 |
|  |  |  |  |
| A2 | CTRL | 72 | 0.51 ± 0.07 |
|  |  | 96 | 0.74 ± 0.09 |
|  | vaLPS | 72 | 0.58 ± 0.14 |
|  |  | 96 | 0.92 ± 0.30 |
|  | *Vang* | 72 | 1.52 ± 0.13 |
|  |  | 96 | 2.18 ± 0.40 |
|  |  |  |  |
| T1 | CTRL | 72 | 0.51 ± 0.17 |
|  |  | 96 | 0.76 ± 0.18 |
|  | vaLPS | 72 | 0.61 ± 0.17 |
|  |  | 96 | 1.11 ± 0.48 |
|  | *Vang* | 72 | 1.50 ± 0.16 |
|  |  | 96 | 2.05 ± 0.49 |
|  |  |  |  |
| T2 | CTRL | 72 | 0.52 ± 0.17 |
|  |  | 96 | 0.76 ± 0.21 |
|  | vaLPS | 72 | 0.68 ± 0.07 |
|  |  | 96 | 1.21 ± 0.46 |
|  | *Vang* | 72 | 1.54 ± 0.23 |
|  |  | 96 | 2.61 ± 0.21 |
|  |  |  |  |
| M1 | CTRL | 72 | 0.48 ± 0.14 |
|  |  | 96 | 0.61 ± 0.07 |
|  | vaLPS | 72 | 0.80 ± 0.03 |
|  |  | 96 | 1.31 ± 0.40 |
|  | *Vang* | 72 | 1.51 ± 0.33 |
|  |  | 96 | 2.53 ± 0.09 |
|  |  |  |  |
| M2 | CTRL | 72 | 0.45 ± 0.11 |
|  |  | 96 | 0.83 ± 0.12 |
|  | vaLPS | 72 | 1.04 ± 0.39 |
|  |  | 96 | 2.68 ± 1.17 |
|  | *Vang* | 72 | 1.44 ± 0.13 |
|  |  | 96 | 2.67 ± 0.32 |

| Multifactorial ANOVA | AA | time | stimulus | AA × time | AA × stimulus | time × stimulus | AA × time × stimulus |
| --- | --- | --- | --- | --- | --- | --- | --- |
| NO (µM) | < 0.001 | < 0.001 | < 0.001 | 0.003 | < 0.001 | ns | ns |

| Table  S1 B | AA | | | | | | | | |  | time | |  | stimulus | | |
| --- | --- | --- | --- | --- | --- | --- | --- | --- | --- | --- | --- | --- | --- | --- | --- | --- |
|  | L-15 | G1 | G2 | A1 | A2 | T1 | T2 | M1 | M2 |  | 72 h | 96 h |  | CTRL | vaLPS | *Vang* |
| NO (µM) | ab | c | ab | a | ab | abc | bc | abc | c |  | x | y |  | * | # | § |

| Table  S1 C | AA × time | | | | | | | | |
| --- | --- | --- | --- | --- | --- | --- | --- | --- | --- |
|  | L-15 | G1 | G2 | A1 | A2 | T1 | T2 | M1 | M2 |
| 72 h | ax | b | ax | a | abx | abx | abx | abx | abx |
| 96 h | aby | bc | bcy | a | aby | aby | abcy | abcy | cy |

| Table  S1 D | AA × stimulus | | | | | | | | |
| --- | --- | --- | --- | --- | --- | --- | --- | --- | --- |
|  | L-15 | G1 | G2 | A1 | A2 | T1 | T2 | M1 | M2 |
| CTRL | *ab | *b | *a | *a | *a | *a | *a | *a | *a |
| vaLPS | *a | *ab | *ab | *ab | *ab | *ab | *ab | #bc | #c |
| *Vang* | # | # | # | # | # | # | # | § | # |

Values represent means ± SD (n = 6 biological replicates). Different symbols stand for statistically significant differences attributed to stimulation. a, b, c and d denote statistically significant differences between AA treatments. x and y stand for significant differences attributed to incubation time. (Multifactorial ANOVA; Tukey post-hoc test; ns: non-significant; P ≤ 0.05).

Table S2 A. Extracellular O_2_^-^ content in the supernatant of European seabass HKL subjected to experimental conditions for 4 h or 24 h.

| AA | stimulus | time | O_2_^-^ (nmol) |
| --- | --- | --- | --- |
| L-15 | CTRL | 4 | 3.26 ± 1.61 |
|  |  | 24 | 1.98 ± 0.53 |
|  | *Vang* | 4 | 2.46 ± 1.12 |
|  |  | 24 | 1.17 ± 0.56 |
|  |  |  |  |
| G1 | CTRL | 4 | 3.19 ± 1.44 |
|  |  | 24 | 2.00 ± 0.67 |
|  | *Vang* | 4 | 2.64 ± 0.86 |
|  |  | 24 | 1.24 ± 0.57 |
|  |  |  |  |
| G2 | CTRL | 4 | 3.07 ± 1.08 |
|  |  | 24 | 2.56 ± 0.20 |
|  | *Vang* | 4 | 2.80 ± 1.30 |
|  |  | 24 | 1.92 ± 1.39 |
|  |  |  |  |
| A1 | CTRL | 4 | 3.82 ± 0.74 |
|  |  | 24 | 2.04 ± 0.44 |
|  | *Vang* | 4 | 2.16 ± 1.08 |
|  |  | 24 | 2.06 ± 1.11 |
|  |  |  |  |
| A2 | CTRL | 4 | 4.24 ± 1.33 |
|  |  | 24 | 2.87 ± 0.76 |
|  | *Vang* | 4 | 3.32 ± 1.05 |
|  |  | 24 | 1.78 ± 1.20 |
|  |  |  |  |
| T1 | CTRL | 4 | 4.71 ± 1.44 |
|  |  | 24 | 2.90 ± 1.16 |
|  | *Vang* | 4 | 3.15 ± 1.34 |
|  |  | 24 | 1.95 ± 1.59 |
|  |  |  |  |
| T2 | CTRL | 4 | 4.23 ± 1.38 |
|  |  | 24 | 3.29 ± 1.49 |
|  | *Vang* | 4 | 3.61 ± 1.27 |
|  |  | 24 | 2.57 ± 1.13 |
|  |  |  |  |
| M1 | CTRL | 4 | 4.80 ± 1.54 |
|  |  | 24 | 3.36 ± 1.30 |
|  | *Vang* | 4 | 3.65 ± 1.24 |
|  |  | 24 | 2.70 ± 0.66 |
|  |  |  |  |
| M2 | CTRL | 4 | 4.87 ± 1.26 |
|  |  | 24 | 3.61 ± 1.04 |
|  | *Vang* | 4 | 3.13 ± 0.75 |
|  |  | 24 | 2.61 ± 1.40 |

| Multifactorial ANOVA | AA | time | stimulus | AA × time | AA × stimulus | time × stimulus | AA × time × stimulus |
| --- | --- | --- | --- | --- | --- | --- | --- |
| Extracellular O_2_^-^ (nmol) | < 0.001 | < 0.001 | < 0.001 | ns | ns | ns | ns |

| Table S2 B | AA | | | | | | | | |  | time | |  | stimulus | |
| --- | --- | --- | --- | --- | --- | --- | --- | --- | --- | --- | --- | --- | --- | --- | --- |
|  | L-15 | G1 | G2 | A1 | A2 | T1 | T2 | M1 | M2 |  | 4 h | 24 h |  | CTRL | *Vang* |
| Extracellular  O_2_^-^ (nmol) | a | a | abc | ab | abcd | abcd | bcd | d | cd |  | y | x |  | # | * |

Values represent means ± SD (n = 6 biological replicates). Different symbols stand for statistically significant differences attributed to stimulation. a, b, c and d denote statistically significant differences between AA treatments. x and y stand for significant differences attributed to time (Multifactorial ANOVA; Tukey post-hoc test; ns: non-significant; P ≤ 0.05).

| AA | Stimulus | Time | ATP (nM) |
| --- | --- | --- | --- |
| L-15 | CTRL | 4 | 3.83 ± 2.96 a |
|  |  | 24 | 9.49 ± 11.53 a |
|  | vaLPS | 4 | 10.86 ± 9.30 a |
|  |  | 24 | 42.25 ± 11.20 |
|  |  |  |  |
| G1 | CTRL | 4 | 6.10 ± 2.67 abx |
|  |  | 24 | 46.29 ± 15.11 abcy |
|  | vaLPS | 4 | 19.86 ± 8.34 ab |
|  |  | 24 | 32.69 ± 16.16 |
|  |  |  |  |
| G2 | CTRL | 4 | 6.40 ± 2.00 abx |
|  |  | 24 | 81.87 ± 46.25 #cy |
|  | vaLPS | 4 | 16.93 ± 3.63 ab |
|  |  | 24 | 32.13 ± 17.64 * |
|  |  |  |  |
| A1 | CTRL | 4 | 14.74 ± 5.52 ab |
|  |  | 24 | 27.93 ± 5.08 ab |
|  | vaLPS | 4 | 27.23 ± 19.83 abcx |
|  |  | 24 | 65.52 ± 27.64 y |
|  |  |  |  |
| A2 | CTRL | 4 | 4.00 ± 1.16 *ab |
|  |  | 24 | 37.80 ± 18.71 ab |
|  | vaLPS | 4 | 56.62 ± 23.07 #c |
|  |  | 24 | 45.71 ± 13.27 |
|  |  |  |  |
| T1 | CTRL | 4 | 69.64 ± 33.68 cy |
|  |  | 24 | 30.64 ± 7.24 abx |
|  | vaLPS | 4 | 49.39 ± 18.86 bc |
|  |  | 24 | 38.78 ± 16.97 |
|  |  |  |  |
| T2 | CTRL | 4 | 40.62 ± 24.51 b |
|  |  | 24 | 48.33 ± 16.55 bc |
|  | vaLPS | 4 | 34.99 ± 22.19 abc |
|  |  | 24 | 33.54 ± 10.66 |
|  |  |  |  |
| M1 | CTRL | 4 | 24.51 ± 10.23 ab |
|  |  | 24 | 62.37 ± 29.20 bc |
|  | vaLPS | 4 | 38.83 ± 15.33 abc |
|  |  | 24 | 37.03 ± 15.29 |
|  |  |  |  |
| M2 | CTRL | 4 | 13.85 ± 12.54 ab |
|  |  | 24 | 31.16 ± 12.55 ab |
|  | vaLPS | 4 | 20.27 ± 4.93 ab |
|  |  | 24 | 39.21 ± 20.74 |

Table S3 A. Extracellular ATP content in the supernatant of European seabass HKL subjected to experimental conditions for 4 h or 24 h.

| Multifactorial ANOVA | AA | time | stimulus | AA × time | AA × stimulus | time × stimulus | AA × time × stimulus |
| --- | --- | --- | --- | --- | --- | --- | --- |
| ATP (nM) | < 0.001 | < 0.001 | ns | < 0.001 | < 0.001 | 0.019 | < 0.001 |

| Table  S3 B | AA | | | | | | | | |  | time | |
| --- | --- | --- | --- | --- | --- | --- | --- | --- | --- | --- | --- | --- |
|  | L-15 | G1 | G2 | A1 | A2 | T1 | T2 | M1 | M2 |  | 4 h | 24 h |
| ATP (nM) | a | a | bcd | bc | bcd | d | cd | cd | ab |  | x | y |

| Table  S3 C | AA × time | | | | | | | | |
| --- | --- | --- | --- | --- | --- | --- | --- | --- | --- |
|  | L-15 | G1 | G2 | A1 | A2 | T1 | T2 | M1 | M2 |
| 4 h | a | abx | abx | abc | bc | dy | cd | bc | bc |
| 24 h | a | abcy | cy | abc | abc | abx | abc | bc | ab |

| Table  S3 D | AA × stimulus | | | | | | | | |
| --- | --- | --- | --- | --- | --- | --- | --- | --- | --- |
|  | L-15 | G1 | G2 | A1 | A2 | T1 | T2 | M1 | M2 |
| CTRL | a | abc | bcd | abc | *abc | d | cd | bcd | ab |
| vaLPS | a | ab | ab | bc | #c | bc | abc | abc | ab |

| Table S3 E | time × stimulus | |
| --- | --- | --- |
|  | 4 h | 24 h |
| CTRL | *x | y |
| vaLPS | #x | y |

Values represent means ± SD (n = 6 biological replicates). Different symbols stand for statistically significant differences attributed to stimulation. a, b, c and d denote statistically significant differences between AA treatments. x and y stand for significant differences attributed to incubation time. (Multifactorial ANOVA; Tukey post-hoc test; ns: non-significant; P ≤ 0.05).

| AA | Stimulus | Time | Arginase activity (U L^-1^) |
| --- | --- | --- | --- |
| L-15 | CTRL | 4 | 1.02 ± 0.78 |
|  |  | 24 | 1.85 ± 0.06 |
|  | *Vang* | 4 | 1.17 ± 0.25 |
|  |  | 24 | 2.20 ± 0.41 |
|  |  |  |  |
| A1 | CTRL | 4 | 1.33 ± 0.63 |
|  |  | 24 | 1.50 ± 0.71 |
|  | *Vang* | 4 | 1.27 ± 0.67 |
|  |  | 24 | 2.05 ± 1.39 |
|  |  |  |  |
| A2 | CTRL | 4 | 1.68 ± 0.72 |
|  |  | 24 | 1.51 ± 0.72 |
|  | *Vang* | 4 | 1.10 ± 0.18 |
|  |  | 24 | 1.63 ± 0.45 |

| Multifactorial ANOVA | [AA] | time | stimulus | [AA] × time | [AA] × stimulus | time × stimulus | [AA] × time × stimulus |
| --- | --- | --- | --- | --- | --- | --- | --- |
| Arginase activity (U L^-1^) | ns | 0.014 | ns | ns | ns | ns | ns |

Table S4 A. Arginase activity in the supernatant of European seabass HKL subjected to experimental conditions for 4 h or 24 h.

| Table S4 B | time | |
| --- | --- | --- |
|  | 4 | 24 |
| Arginase activity (U L^-1^) | x | y |

Values represent means ± SD (n = 6 biological replicates). x and y stand for significant differences attributed to incubation time. (Multifactorial ANOVA; Tukey post-hoc test; ns: non-significant; P ≤ 0.05).

Table S5 A. Expression patterns of genes involved in the immune response and polyamine biosynthesis of head-kidney leucocytes subjected to the experimental conditions for 4 and 24 h.

|  |  |  | Gene Expression (Fold Change) | | | | | | | | | | | | | | | | | | | | |
| --- | --- | --- | --- | --- | --- | --- | --- | --- | --- | --- | --- | --- | --- | --- | --- | --- | --- | --- | --- | --- | --- | --- | --- |
| Treatments | Stimulus | Time | *il1β* | | | *cox2* | | | *mif* | | | *tgfβ* | | | *amd* | | | *odc* | | | *sat* | | |
| L-15 | CTRL | 4 | 0.78 | ± | 0.40 | 0.57 | ± | 0.22 | 1.00 | ± | 1.20 | 0.74 | ± | 0.65 | 0.80 | ± | 0.23 | 0.97 | ± | 0.23 | 0.77 | ± | 0.09 |
|  |  | 24 | 0.97 | ± | 1.03 | 2.69 | ± | 3.54 | 0.30 | ± | 0.27 | 0.49 | ± | 0.31 | 1.42 | ± | 0.33 | 4.73 | ± | 0.70 | 0.56 | ± | 0.13 *a |
|  | vaLPS | 4 | 9.30 | ± | 6.93 | 1.54 | ± | 1.02 | 0.64 | ± | 0.54 ab | 0.88 | ± | 0.31 | 1.51 | ± | 0.25 | 1.72 | ± | 1.60 | 0.51 | ± | 0.20 abx |
|  |  | 24 | 0.24 | ± | 0.14 | 0.58 | ± | 0.52 | 1.74 | ± | 1.40 | 0.76 | ± | 0.33 | 1.34 | ± | 0.21 | 0.56 | ± | 0.17 | 2.01 | ± | 0.72 #aby |
|  |  |  |  |  |  |  |  |  |  |  |  |  |  |  |  |  |  |  |  |  |  |  |  |
| G1 | CTRL | 4 | 0.34 | ± | 0.19 | 1.46 | ± | 0.93 | 0.84 | ± | 0.41 | 1.43 | ± | 0.20 | 0.85 | ± | 0.09 | 0.36 | ± | 0.07 | 0.52 | ± | 0.03 |
|  |  | 24 | 1.16 | ± | 1.41 | 4.19 | ± | 5.28 | 4.13 | ± | 1.82 | 1.17 | ± | 0.14 | 0.54 | ± | 0.16 | 0.06 | ± | 0.01 | 1.38 | ± | 0.39 ab |
|  | vaLPS | 4 | 10.56 | ± | 4.46 | 12.17 | ± | 6.14 | 1.52 | ± | 0.51 b | 1.14 | ± | 0.24 | 0.61 | ± | 0.13 | 0.11 | ± | 0.06 | 0.59 | ± | 0.10 ab |
|  |  | 24 | 0.37 | ± | 0.11 | 1.02 | ± | 0.64 | 3.23 | ± | 0.48 | 0.93 | ± | 0.18 | 0.53 | ± | 0.17 | 0.12 | ± | 0.06 | 1.01 | ± | 0.30 a |
|  |  |  |  |  |  |  |  |  |  |  |  |  |  |  |  |  |  |  |  |  |  |  |  |
| G2 | CTRL | 4 | 1.31 | ± | 0.16 | 4.28 | ± | 1.26 | 0.79 | ± | 0.26 | 1.08 | ± | 0.50 | 1.23 | ± | 0.64 | 0.22 | ± | 0.09 | 0.57 | ± | 0.13 |
|  |  | 24 | 0.59 | ± | 0.43 | 1.86 | ± | 1.53 | 1.83 | ± | 0.44 | 0.72 | ± | 0.21 | 0.48 | ± | 0.18 | 0.09 | ± | 0.04 | 1.32 | ± | 0.23 ab |
|  | vaLPS | 4 | 7.54 | ± | 6.09 | 7.31 | ± | 3.71 | 1.21 | ± | 0.21 b | 0.79 | ± | 0.24 | 1.01 | ± | 0.24 | 0.25 | ± | 0.11 | 0.49 | ± | 0.13 ab |
|  |  | 24 | 0.42 | ± | 0.26 | 1.89 | ± | 1.02 | 1.04 | ± | 0.46 | 0.80 | ± | 0.31 | 0.64 | ± | 0.03 | 0.11 | ± | 0.03 | 1.33 | ± | 0.17 ab |
|  |  |  |  |  |  |  |  |  |  |  |  |  |  |  |  |  |  |  |  |  |  |  |  |
| A1 | CTRL | 4 | 0.80 | ± | 0.56 | 0.68 | ± | 0.34 | 1.02 | ± | 0.81 | 0.75 | ± | 0.42 | 0.50 | ± | 0.14 | 0.70 | ± | 0.35 | 0.56 | ± | 0.20 |
|  |  | 24 | 0.08 | ± | 0.11 | 0.37 | ± | 0.45 | 0.95 | ± | 1.40 | 0.09 | ± | 0.09 | 0.49 | ± | 0.14 | 0.31 | ± | 0.22 | 0.64 | ± | 0.24 *ab |
|  | vaLPS | 4 | 7.13 | ± | 4.04 | 1.79 | ± | 1.00 | 0.41 | ± | 0.36 b | 0.51 | ± | 0.46 | 0.61 | ± | 0.26 | 1.21 | ± | 0.92 | 0.84 | ± | 0.15 abx |
|  |  | 24 | 0.26 | ± | 0.18 | 0.17 | ± | 0.14 | 1.38 | ± | 1.30 | 1.12 | ± | 0.99 | 1.04 | ± | 0.71 | 0.36 | ± | 0.39 | 2.39 | ± | 1.56 #aby |
|  |  |  |  |  |  |  |  |  |  |  |  |  |  |  |  |  |  |  |  |  |  |  |  |
| A2 | CTRL | 4 | 0.53 | ± | 0.27 | 0.42 | ± | 0.42 | 0.07 | ± | 0.08 | 0.25 | ± | 0.10 | 0.72 | ± | 0.08 | 1.07 | ± | 0.92 | 0.68 | ± | 0.17 |
|  |  | 24 | 0.07 | ± | 0.06 | 0.04 | ± | 0.04 | 0.50 | ± | 0.51 | 0.58 | ± | 0.54 | 0.64 | ± | 0.18 | 0.46 | ± | 0.27 | 0.82 | ± | 0.32 *ab |
|  | vaLPS | 4 | 5.09 | ± | 3.05 | 0.89 | ± | 0.78 | 0.25 | ± | 0.22 ab | 0.43 | ± | 0.25 | 0.87 | ± | 0.21 | 0.64 | ± | 0.40 | 0.87 | ± | 0.23 bx |
|  |  | 24 | 0.26 | ± | 0.17 | 0.20 | ± | 0.10 | 1.05 | ± | 0.85 | 0.85 | ± | 0.13 | 1.74 | ± | 0.83 | 0.31 | ± | 0.19 | 2.87 | ± | 0.96 #by |
|  |  |  |  |  |  |  |  |  |  |  |  |  |  |  |  |  |  |  |  |  |  |  |  |
| T1 | CTRL | 4 | 0.74 | ± | 0.15 | 0.84 | ± | 0.02 | 0.16 | ± | 0.09 | 0.69 | ± | 0.34 | 0.94 | ± | 0.45 | 0.43 | ± | 0.29 | 0.46 | ± | 0.05 |
|  |  | 24 | 0.09 | ± | 0.05 | 0.31 | ± | 0.12 | 2.16 | ± | 1.36 | 0.85 | ± | 0.14 | 0.54 | ± | 0.01 | 0.13 | ± | 0.05 | 1.02 | ± | 0.14 ab |
|  | vaLPS | 4 | 7.09 | ± | 4.93 | 3.65 | ± | 1.10 | 0.52 | ± | 0.07 ab | 0.82 | ± | 0.30 | 0.79 | ± | 0.37 | 0.28 | ± | 0.22 | 0.45 | ± | 0.23 abx |
|  |  | 24 | 0.29 | ± | 0.11 | 2.68 | ± | 0.33 | 2.24 | ± | 1.88 | 1.13 | ± | 0.11 | 0.82 | ± | 0.17 | 0.13 | ± | 0.01 | 2.96 | ± | 1.51 by |
|  |  |  |  |  |  |  |  |  |  |  |  |  |  |  |  |  |  |  |  |  |  |  |  |
| T2 | CTRL | 4 | 0.60 | ± | 0.44 | 0.52 | ± | 0.40 | 0.58 | ± | 0.67 | 0.44 | ± | 0.52 | 0.56 | ± | 0.21 | 1.16 | ± | 0.89 | 0.66 | ± | 0.23 x |
|  |  | 24 | 0.16 | ± | 0.13 | 0.06 | ± | 0.04 | 0.91 | ± | 0.89 | 0.93 | ± | 0.41 | 0.55 | ± | 0.10 | 0.40 | ± | 0.09 | 1.60 | ± | 0.41 by |
|  | vaLPS | 4 | 5.07 | ± | 4.12 | 1.49 | ± | 0.92 | 0.61 | ± | 0.66 ab | 0.37 | ± | 0.26 | 0.66 | ± | 0.29 | 1.22 | ± | 0.77 | 0.38 | ± | 0.17 ax |
|  |  | 24 | 0.18 | ± | 0.16 | 0.24 | ± | 0.21 | 1.29 | ± | 1.30 | 0.70 | ± | 0.27 | 0.92 | ± | 0.20 | 0.27 | ± | 0.19 | 1.84 | ± | 0.60 aby |
|  |  |  |  |  |  |  |  |  |  |  |  |  |  |  |  |  |  |  |  |  |  |  |  |
| M1 | CTRL | 4 | 0.53 | ± | 0.03 | 1.66 | ± | 0.75 | 0.35 | ± | 0.12 | 0.54 | ± | 0.44 | 1.03 | ± | 0.33 | 0.29 | ± | 0.07 | 0.43 | ± | 0.03 x |
|  |  | 24 | 0.09 | ± | 0.07 | 0.52 | ± | 0.33 | 4.31 | ± | 3.36 | 0.80 | ± | 0.01 | 0.48 | ± | 0.15 | 0.07 | ± | 0.02 | 1.28 | ± | 0.28 aby |
|  | vaLPS | 4 | 8.14 | ± | 2.02 | 3.68 | ± | 0.48 | 0.25 | ± | 0.17 ab | 0.44 | ± | 0.24 | 1.02 | ± | 0.38 | 0.53 | ± | 0.19 | 1.06 | ± | 0.30 b |
|  |  | 24 | 0.36 | ± | 0.23 | 2.79 | ± | 0.23 | 3.90 | ± | 3.16 | 0.97 | ± | 0.45 | 0.65 | ± | 0.24 | 0.10 | ± | 0.02 | 1.69 | ± | 0.35 ab |
|  |  |  |  |  |  |  |  |  |  |  |  |  |  |  |  |  |  |  |  |  |  |  |  |
| M2 | CTRL | 4 | 0.45 | ± | 0.13 | 0.25 | ± | 0.21 | 0.42 | ± | 0.44 | 0.37 | ± | 0.44 | 0.63 | ± | 0.11 | 0.49 | ± | 0.32 | 0.69 | ± | 0.53 |
|  |  | 24 | 0.04 | ± | 0.04 | 0.21 | ± | 0.39 | 0.77 | ± | 0.90 | 0.39 | ± | 0.49 | 0.74 | ± | 0.07 | 0.68 | ± | 0.52 | 1.00 | ± | 0.29 ab |
|  | vaLPS | 4 | 9.08 | ± | 2.62 | 0.80 | ± | 0.88 | 0.24 | ± | 0.38 ax | 0.14 | ± | 0.13 | 0.52 | ± | 0.19 | 0.40 | ± | 0.62 | 0.48 | ± | 0.14 abx |
|  |  | 24 | 0.30 | ± | 0.17 | 0.28 | ± | 0.28 | 2.09 | ± | 1.13 y | 1.18 | ± | 0.71 | 2.64 | ± | 2.49 | 0.19 | ± | 0.11 | 1.84 | ± | 0.64 y |

| Multifactorial ANOVA | AA | time | stimulus | AA × time | AA × stimulus | time × stimulus | AA × time  × stimulus |
| --- | --- | --- | --- | --- | --- | --- | --- |
| *il1β* | 0.011 | < 0.001 | < 0.001 | ns | 0.026 | < 0.001 | ns |
| *cox2* | < 0.001 | < 0.001 | < 0.001 | ns | ns | ns | ns |
| *mif* | 0.002 | < 0.001 | ns | ns | ns | ns | 0.037 |
| *tgfβ* | < 0.001 | 0.030 | ns | 0.028 | ns | 0.009 | ns |
| *amd* | < 0.001 | ns | 0.002 | < 0.001 | ns | 0.006 | ns |
| *odc* | < 0.001 | < 0.001 | 0.025 | 0.004 | 0.031 | ns | ns |
| *sat* | ns | < 0.001 | < 0.001 | 0.004 | < 0.001 | < 0.001 | < 0.001 |

| Table S5 B |  | | | | | | | | |  |  | |  |  | |  | AA × time | | | | | | | | | | | | | | | | | | |
| --- | --- | --- | --- | --- | --- | --- | --- | --- | --- | --- | --- | --- | --- | --- | --- | --- | --- | --- | --- | --- | --- | --- | --- | --- | --- | --- | --- | --- | --- | --- | --- | --- | --- | --- | --- |
|  | AA | | | | | | | | |  | time | |  | stimulus | |  | 4 h | | | | | | | | |  | 24 h | | | | | | | | |
|  | L-15 | G1 | G2 | A1 | A2 | T1 | T2 | M1 | M2 |  | 4 h | 24 h |  | CTRL | vaLPS |  | L-15 | G1 | G2 | A1 | A2 | T1 | T2 | M1 | M2 |  | L-15 | G1 | G2 | A1 | A2 | T1 | T2 | M1 | M2 |
| *il1β* | b | ab | ab | ab | ab | ab | a | ab | ab |  | y | x |  | * | # |  |  |  |  |  |  |  |  |  |  |  |  |  |  |  |  |  |  |  |  |
| *cox2* | bc | cd | d | ab | a | cd | ab | cd | a |  | y | x |  | * | # |  |  |  |  |  |  |  |  |  |  |  |  |  |  |  |  |  |  |  |  |
| *mif* | ab | b | ab | ab | a | ab | ab | ab | a |  | x | y |  |  |  |  |  |  |  |  |  |  |  |  |  |  |  |  |  |  |  |  |  |  |  |
| *tgfβ* | ab | b | ab | a | a | ab | a | a | a |  | x | y |  |  |  |  | abc | c | bc | ab | ab | abc | ab | ab | a |  |  |  |  |  |  |  |  |  |  |
| *amd* | b | a | ab | a | ab | a | a | a | ab |  |  |  |  | * | # |  | ab | a | b | ab | ab | ab | ab | a | abx |  | b | a | a | a | ab | ab | ab | a | by |
| *odc* | d | a | ab | bcd | bcd | abc | cd | ab | abc |  | y | x |  | # | * |  | ab | ab | ab | b | ab | ab | b | ab | a |  | b | a | a | a | a | a | ab | a | a |
| *sat* |  |  |  |  |  |  |  |  |  |  | x | y |  | * | # |  | x | x | x | x | x | x | x | x | x |  | y | y | y | y | y | y | y | y | y |

| Table  S5 C | time × stimulus | | | |  | AA × stimulus | | | | | | | | | | | | | | | | | | |
| --- | --- | --- | --- | --- | --- | --- | --- | --- | --- | --- | --- | --- | --- | --- | --- | --- | --- | --- | --- | --- | --- | --- | --- | --- |
|  | 4 | | 24 | |  | CTRL | | | | | | | | |  | vaLPS | | | | | | | | |
|  | CTRL | vaLPS | CTRL | vaLPS |  | L-15 | G1 | G2 | A1 | A2 | T1 | T2 | M1 | M2 |  | L-15 | G1 | G2 | A1 | A2 | T1 | T2 | M1 | M2 |
| *il1β* | *x | #y | #y | *x |  | b | ab | ab | *ab | *a | ab | ab | *ab | Aa |  |  |  |  | # | # |  |  | # | # |
| *cox2* |  |  |  |  |  |  |  |  |  |  |  |  |  |  |  |  |  |  |  |  |  |  |  |  |
| *mif* |  |  |  |  |  |  |  |  |  |  |  |  |  |  |  |  |  |  |  |  |  |  |  |  |
| *tgfβ* |  | x | * | #y |  |  |  |  |  |  |  |  |  |  |  |  |  |  |  |  |  |  |  |  |
| *amd* | y |  | *x | # |  |  |  |  |  |  |  |  |  |  |  |  |  |  |  |  |  |  |  |  |
| *odc* |  |  |  |  |  | #b | a | a | a | a | a | a | a | a |  | *b | a | a | ab | ab | a | ab | a | a |
| *sat* | x | x | *y | #y |  |  |  |  | * | * |  |  |  |  |  | abc | ab | a | #bc | #c | abc | a | bc | abc |

Values represent means ± SD (n = 6). Different symbols stand for statistically significant differences attributed to stimulation. a, b, c and d denote statistically significant differences between AA treatments. x and y stand for statistically significant differences attributed to incubation time. (Multifactorial ANOVA; Tukey post-hoc test; ns: non-significant; P ≤ 0.05).
